# Supplementary material for: HIVprotI: an integrated web based platform for prediction and design of HIV proteins inhibitors
Source: J Cheminform. 2018 Mar 9;10:12. doi: 10.1186/s13321-018-0266-y (PMC5845081; doi:10.1186/s13321-018-0266-y)
Supplement: Supplementary file 2 — Additional file 2. Source code of HIVProtI web server. [file 13321_2018_266_MOESM2_ESM.zip › HIVprotI_Source-code/hivprotI/weka-3-6-6/doc/weka/associations/SingleAssociatorEnhancer.html]

SingleAssociatorEnhancer


---


|  |  |  |
| --- | --- | --- |
| **Overview** \| **Package** \| **Class** \| **Tree** \| **Deprecated** \| **Index** \| **Help** \| **Weka's home** | |  |
| **PREV CLASS**   **NEXT CLASS** | **FRAMES**    **NO FRAMES**     **All Classes** |  |
| SUMMARY: NESTED \| FIELD \| CONSTR \| METHOD | DETAIL: FIELD \| CONSTR \| METHOD |  |


---


## weka.associations Class SingleAssociatorEnhancer

```
java.lang.Object
  weka.associations.AbstractAssociator
      weka.associations.SingleAssociatorEnhancer
```

**All Implemented Interfaces:**: java.io.Serializable, java.lang.Cloneable, Associator, CapabilitiesHandler, OptionHandler, RevisionHandler

**Direct Known Subclasses:**: FilteredAssociator

---

``` public abstract class SingleAssociatorEnhancer extends AbstractAssociator implements OptionHandler ```

Abstract utility class for handling settings common to meta
associators that use a single base associator.

**Version:**
:   $Revision: 5504 $

**Author:**
:   Eibe Frank (eibe@cs.waikato.ac.nz), FracPete (fracpete at waikato dot ac dot nz)

**See Also:**: Serialized Form

---

**Constructor Summary**

`SingleAssociatorEnhancer()`


| **Method Summary** | |
| --- | --- |
| `java.lang.String` | `associatorTipText()`             Returns the tip text for this property |
| `Associator` | `getAssociator()`             Get the associator used as the base associator. |
| `Capabilities` | `getCapabilities()`             Returns default capabilities of the base associator. |
| `java.lang.String[]` | `getOptions()`             Gets the current settings of the associator. |
| `java.util.Enumeration` | `listOptions()`             Returns an enumeration describing the available options. |
| `void` | `setAssociator(Associator value)`             Set the base associator. |
| `void` | `setOptions(java.lang.String[] options)`             Parses a given list of options. |

**Methods inherited from class weka.associations.AbstractAssociator**

`forName, getRevision, makeCopies, makeCopy`

**Methods inherited from class java.lang.Object**

`equals, getClass, hashCode, notify, notifyAll, toString, wait, wait, wait`

**Methods inherited from interface weka.associations.Associator**

`buildAssociations`

**Constructor Detail**

### SingleAssociatorEnhancer

```
public SingleAssociatorEnhancer()
```


**Method Detail**

### listOptions

```
public java.util.Enumeration listOptions()
```

:   Returns an enumeration describing the available options.

    :   **Specified by:**: `listOptions` in interface `OptionHandler`
    :   **Returns:**: an enumeration of all the available options.

---


### setOptions

```
public void setOptions(java.lang.String[] options)
                throws java.lang.Exception
```

:   Parses a given list of options. Valid options are:

    -W classname   
    Specify the full class name of the base associator.

    Options after -- are passed to the designated associator.

    :   **Specified by:**: `setOptions` in interface `OptionHandler`
    :   **Parameters:**: `options` - the list of options as an array of strings **Throws:**: `java.lang.Exception` - if an option is not supported

---


### getOptions

```
public java.lang.String[] getOptions()
```

:   Gets the current settings of the associator.

    :   **Specified by:**: `getOptions` in interface `OptionHandler`
    :   **Returns:**: an array of strings suitable for passing to setOptions

---


### associatorTipText

```
public java.lang.String associatorTipText()
```

:   Returns the tip text for this property

    :   **Returns:**: tip text for this property suitable for displaying in the explorer/experimenter gui

---


### setAssociator

```
public void setAssociator(Associator value)
```

:   Set the base associator.

    :   **Parameters:**: `value` - the associator to use.

---


### getAssociator

```
public Associator getAssociator()
```

:   Get the associator used as the base associator.

    :   **Returns:**: the currently used associator

---


### getCapabilities

```
public Capabilities getCapabilities()
```

:   Returns default capabilities of the base associator.

    :   **Specified by:**: `getCapabilities` in interface `Associator` **Specified by:**: `getCapabilities` in interface `CapabilitiesHandler` **Overrides:**: `getCapabilities` in class `AbstractAssociator`
    :   **Returns:**: the capabilities of the base associator **See Also:**: `Capabilities`


---


|  |  |  |
| --- | --- | --- |
| **Overview** \| **Package** \| **Class** \| **Tree** \| **Deprecated** \| **Index** \| **Help** \| **Weka's home** | |  |
| **PREV CLASS**   **NEXT CLASS** | **FRAMES**    **NO FRAMES**     **All Classes** |  |
| SUMMARY: NESTED \| FIELD \| CONSTR \| METHOD | DETAIL: FIELD \| CONSTR \| METHOD |  |


---
